# Surface-displayed silicatein-α enzyme in bioengineered *E. coli* enables biocementation and silica mineralization

**DOI:** 10.3389/fsysb.2024.1377188

**Published:** 2024-05-30

**Authors:** Toriana N. Vigil, Nikolas K. Schwendeman, Melanie L. M. Grogger, Victoria L. Morrison, Margaret C. Warner, Nathaniel B. Bone, Morgan T. Vance, David C. Morris, Kristi McElmurry, Bryan W. Berger, J. Jordan Steel

**Affiliations:** ^1^ Department of Chemical Engineering, University of Virginia, Charlottesville, VA, United States; ^2^ Department of Biology, United States Air Force Academy, Colorado Springs, CO, United States; ^3^ Life Sciences Research Center, United States Air Force Academy, Colorado Springs, CO, United States

**Keywords:** biocementation, biosilicification, biomineralization, silicatein-α, biotechnology

## Abstract

Biocementation is an exciting biomanufacturing alternative to common cement, which is a significant contributor of CO_2_ greenhouse gas production. In nature biocementation processes are usually modulated via ureolytic microbes, such as *Sporosarcina pasteurii,* precipitating calcium carbonate to cement particles together, but these ureolytic reactions also produce ammonium and carbonate byproducts, which may have detrimental effects on the environment. As an alternative approach, this work examines biosilicification via surface-displayed silicatein-α in bio-engineered *E. coli* as an *in vivo* biocementation strategy. The surface-display of silicatein-α with ice nucleation protein is a novel protein fusion combination that effectively enables biosilicification, which is the polymerization of silica species in solution, from the surface of *E. coli* bacterial cells. Biosilicification with silicatein-α produces biocementation products with comparable compressive strength as *S. pasteurii.* This biosilicification approach takes advantage of the high silica content found naturally in sand and does not produce the ammonium and carbonate byproducts of ureolytic bacteria, making this a more environmentally friendly biocementation strategy.

## Introduction

Cement is a key building block for most structures—ranging from basic homes, to sky-scraper office buildings, to timeless monuments. Unfortunately, cement is also a major contributor of man-made CO_2_ production and a tremendous use of natural resources, resulting in an environmental burden (Gartner and Macphee, 2011; Iqbal et al., 2021). One alternative to man-made cement is microbially induced calcite precipitation (MICP), more commonly known as biocementation. Biocementation usually takes advantage of natural ureolytic and nitrification processes in microbes to precipitate large particle aggregates and can be considered biomanufacturing of cement (Zuo et al., 2023). In the most common usage of MICP, calcium carbonate is precipitated, forming bridges between smaller particles and “cementing” them together. Biocementation has many applications, including use as a building block for structures, soil stabilization and erosion prevention, and dust mitigation; these functions also make it a prime candidate for future space applications on the moon or Mars (Castro-Alonso et al., 2019; Erdmann and Strieth, 2023; Zuo et al., 2023).

The bacteria *Sporosarcina pasteurii* is frequently used in MICP for its high intrinsic urease activity: breaking down urea and producing ammonia and carbonate (Murugan et al., 2021; Erdmann and Strieth, 2023). Carbonate ions then react with exogenous calcium to form precipitated calcium carbonate, which attaches to nearby particles and can link particles together, effectively increasing aggregate size. While there are a significant number of studies using *S. pasteurii* and MICP, there are two important drawbacks to consider: 1) there is relatively low abundance of calcium in soil and 2) the ammonium and carbonate by-products may have potential adverse environmental impacts (Lee et al., 2019; Gowthaman et al., 2022). MICP with *S. pasteurii* is most effective with 22% w/v calcium content; however, the average calcium content in soil is less than 1.5%, thus necessitating the addition of additional calcium as a reagent for biocementation (Shacklette and Boerngen, 1984; Erdmann and Strieth, 2023). Furthermore, while the production of ammonium and carbonate are crucial for MICP with *S. pasteurii*, these chemicals remain in the environment as pollutants (Yu et al., 2021). An overabundance of ammonium and carbonate in the environment can lead to harmful outcomes, such as algal blooms or local acidification (J. Gao et al., 2020; Karadag et al., 2006; Su et al., 2022). While biomanufacturing cement with MICP may be more eco-friendly than traditional cement production, *S. pasteurii* and ureolytic MICP also have negative side effects such as the buildup of ammonium and carbonate. Here we propose biocementation via biosilicification with surface-displayed silicatein-α in bio-engineered *Escherichia coli* as a more sustainable alternative.

**FIGURE 1:**
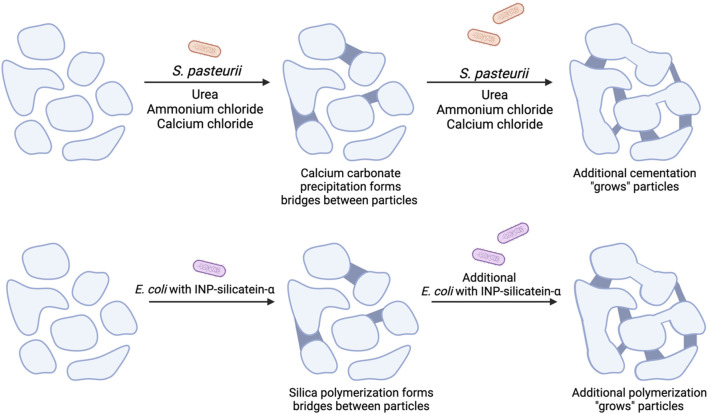
A comparison of MICP with proposed mechanism for biocementation via biosilicification with *E. coli* INP-silicatein-α. Soil particles are initially unbound and have minimal compressive strength. (Top) MICP with the addition of *S. pasteurii,* urea, ammonium chloride, and calcium chloride leads to calcium carbonate precipitation, cementing particles together. Additional treatments lead to additional particle “growth.” (Bottom) Addition of *E. coli* INP-silicatein-α leads to the polymerization of silica species, forming bridges between particles and “growing” aggregate size. Further treatments strengthen these connections by inducing further polymerization.

Biocementation via biosilicification may eliminate the requirement for added reagents such as calcium, as silica is present at approximately 30% w/v in standard soil and sand (Shacklette and Boerngen, 1984; Churchman and Lowe, 2012; Cherian and Arnepalli, 2015). Silicatein-α is a biomineralization enzyme that naturally performs silica polymerization in marine sponges (Shimizu et al., 1998). A proposed comparison between traditional MICP and the biosilicification biocementation strategy is shown in Figure 1. Recent work by Gao, *et al.* highlights silicatein-α precipitation of calcium species in enriched CO_2_ and calcium reagent conditions (K. Gao et al., 2024). Work by Wang *et al.* showed that surface-displayed silicatein in yeast resulted in the formation of a cross-linked matrix of biosilica with embedded yeast cells, supporting our supposition that silicification and not calcium carbonate production is occurring (Wang et al., 2020). The combination of silica and calcium precipitation may lead to enhanced strength in biocementation products and silicatein-α may have a dual use for catalyzing both reactions (K. Gao et al., 2024; Kellermeier et al., 2012). Furthermore, implementing this biosilicification strategy in engineered *E. coli* rather than *S. pasteurii* allows for future genetic engineering for silica pathway optimization. In this work we show surface-display of silicatein-α in *E. coli* enables biocementation via biosilicification in an easily genetically-modifiable system with limited protein processing and purification steps.

## Materials and methods

### Plasmid preparation

INP-silicatein-α (INP accession #Q33479.1, silicatein-α accession #CDO33960.1) was cloned into pET-28a (+) plasmid at with standard molecular biology techniques with BamHI and XhoI. INP-silicatein-α pET-28a (+) was transformed into BL21 (DE3) *E. coli* through electroporation of electrocompetent cells. We used a T7 RNA polymerase and isopropyl ß-D-1-thiogalactopyranoside (IPTG) induction system for the expression of the INP-silicatein-α protein. To induce INP- silicatein-α expression, starter cultures grown overnight were spun in a centrifuge at 3,000 rcf for 10 min. The supernatant was removed, and the pellet was resuspended in terrific broth with kanamycin and 10 glass disruption beads for aeration. The bacterial culture was then shaken at 250 rpm at 37°C until the culture reached an OD600 of 0.60. At the desired OD600 0.1 mM IPTG was introduced to the culture for induction of the INPsilicatein-α protein. The culture was then left shaking at room temperature overnight.

### SDS-PAGE

*E. coli* INP-silicatein-α cell culture was pelleted via centrifugation, then resuspended in sonication lysis buffer (5 mL/1 g cell pellet) consisting of glycerol (99+%) (5% v/v), tris HCl (36 mM), tris base (20 mM), NaCl (100 mM), and imidazole (5 mM). The sample was sonicated via micro-tip sonification at 20% amplitude for 20 min in on/off intervals of 20 s on ice. Following lysis, the lysate was clarified via centrifugation at 10,000 rcf for 10 min. A sample of clarified lysate was then denatured using a thermocycler at 95°C. A 10% Mini-PROTEAN TGX Precast Protein Gel was loaded with serial dilutions of lysed *E. coli* INP- silicatein-α and Laemmli buffer containing ß-mercaptoethanol totaling to 20 µL in each well. One well was loaded with a Bio-Rad Precision Plus Protein ladder. After running the gel at 150 V for 50 min, the gel was fixed with a 50% H_2_O, 40% methanol, and 10% acetic acid solution. The gel was then stained with Bio-Safe™ Coomassie Stain, destained with DI H_2_O, and an image was captured using a Bio-Rad Gel Doc XR+ Gel Documentation System.

### Western blot

Following the SDS-PAGE procedure, the proteins were then transferred to nitrocellulose through a Bio-Rad Criterion Blotter apparatus. The nitrocellulose was incubated in 1x EveryBlot Blocking Buffer (BioRad) for at least 5 min. Then anti-penta-his conjugated HRP antibody (BioRad) diluted 1/1000 was added and incubated for 1 h. The membrane was rinsed gently with 0.1% Tween 20 tris-buffered saline for 3 min and treated with ECl solution (Amersham). Membrane was then imaged with chemiluminescence.

### Immunocytochemistry

After protein expression, the culture was spun down at 3,000 rcf for 10 min. The pellet was then washed twice with tris-buffered saline and resuspended in 100% ice cold methanol, a nonpermeabilizing fixative. After incubating in methanol on ice for 10 min, 100 µL were dropped on a slide and dried. Anti-silicatein-α rabbit unconjugated primary antibody (antibodies.com A81861) diluted in 1/1,000 EveryBlot blocking buffer (BioRad) was added. Slides were then transferred to a humidifying chamber at 4°C overnight. The next day, the slides were washed with tris-buffered saline and anti-rabbit goat AlexaFluor 488 secondary antibody (Invitrogen A27034) diluted 1/10,000 in EveryBlot blocking buffer (BioRad) was added. Slides were incubated for at 37°C 1 h, washed again with tris-buffered saline, and a coverslip was added with ProLong Gold Antifade Mountant with DNA Stain DAPI (ThermoFisher Scientific P3693). The slides were imaged on a Keyence BZ-X810 Fluorescence Microscope with a ×60 objective lens.

### *In vivo* biomineralization

5 mL of induced INP-silicatein-α in *E. coli* cultures was spun down at 3,000 rcf for 10 min. The cell pellet was resuspended in 25 mL of sterile, DI H_2_O in a 125 mL flask. A final concentration of 2 mM sodium orthosilicate was added. Mixture was incubated overnight at room temperature shaking. A WT BL21 *E. coli* and sodium orthosilicate only control were included. After 24 h, cells were removed from solution via centrifugation at 1,000 × g for 10 min. The supernatant was ultracentrifuged in 5 mL increments at 50,000 × g for 45 min to precipitate silica biomineralization products.

### Silicomolybdate assay

Following ultracentrifugation, samples were dried and resuspended in 0.2 M NaOH. Samples were then adjusted to pH 1.6–1.9 with 2 M HCl. After recording total sample volume, samples were transferred to 96-well plate in 200 µL increments. 15 μL of 5% w/v ammonium molybdate was added to each well then incubated at RT for 15 min 15 μL of 0.1% w/v ascorbic acid and 15 µL of 0.1% w/v oxalic acid were added to each well and incubated at RT for 2 h. Absorbance was read at 820 nm on BioTek Syngergy Neo2 plate reader. Nanograms of silica were calculated via Beer’s Law with extinction coefficient 44,710 mol^−1^ cm^−1^ as derived previously (Coradin, T., Eglin, D., Livage, 2004).

### *S. pasteurii* growth

*Sporosarcina pasteurii* (ATCC 11859/DSM 33) cultures were revived from glycerol stocks (5–10 µL) and resuspended with 5 mls of Bacto™ Brain Heart Infusion supplemented with urea (0.3 M). The cultures were grown at room temperature (25°C) overnight (12–16 hrs), shaking.

### Brick construction

In order to construct biocemented bricks, Sandtastik Sparkling White Play Sand (Flinn Scientific #AP9567) was poured into a 3-inch (height) by 1.5-inch (diameter) cylindrical mold lined with stainless steel mesh. 40 mL of cell culture (*S. pasteurii* or *E. coli* INP-silicatein-α) was added dropwise via serological pipette on top of the sand. After waiting 45 min for the culture to fully percolate through the mold, 80 mL of cementation solution was added dropwise via serological pipette. Cementation solution contains urea (0.3 M), ammonium chloride (0.2 M), and calcium chloride dihydrate (0.9 M). 30 min after adding the cementation solution, the next round of biocementation was initiated by dripping 40 mL of cell culture on top of the sand. Three total rounds were completed for each brick. Bricks were left to dry in their mold for 24 h and then removed from the mold and left to cure for 21 days prior to running the unconfined compression test. (Note: although cementation solution is not necessary for silicification with *E. coli* INP-silicatein-α, cementation was applied to these bricks to ensure consistency across experiments and effectively compare the role of *S. pasteurii* and *E. coli* INP-silicatein-α.)

### 3D printing for brick molds

We designed and created the 3-inch (height) by 1.5-inch (diameter) cylindrical molds using a Stratasys Object30 V5 Pro 3D Printer. SolidWorks software was used to design the molds and create a CAD file. Stratasys’ Rigur™ (RGD450) material was used for the molds. After printing, molds were water blasted to remove any structural print residue. Molds were dried for at least 24 h before use for biocementation. Our 3D printed mold design is available upon request to corresponding author.

### Unconfined compression tests

To test the compressive strength of the biocemented bricks, each brick was crushed with an ELE International Versa-Loader (model: 25-3525/02) and a model 20210-500 Type S load cell. [Unconfined Compression Test Digital Readout Set, English 110 vAC (271121/02)]. Each brick was placed alone on the lower loading plate, oriented for axial loading with the circular faces flush against the loading plates. Once bricks were placed on the lower loading plate, about 1 pound of pressure was applied to the brick to secure it in place against both upper and lower loading plates. The digital readout was then tared and the test was commenced. Increasing load was continuously applied [approximately 0.08” (2.032 mm) per minute) until complete structural failure of the brick was achieved, which is detected by the instrument as a sudden decrease in compressive load. The highest compressive load observed in the trial was recorded.

## Results

For effective *in vivo* biosilicification, we designed a surface-display system with silicatein-α expression in *E. coli*. Previous work by Curnow, et al. (2005) utilized a silicatein-α fusion with the outer membrane protein OmpA for the synthesis of titanium phosphates (Curnow et al., 2005; 2006), but did not attempt biocementation. To our knowledge, there have been no other reports of surface-display with silicatein-α for *in vivo* biomineralization. A comparative study of cell-surface display systems by Nicchi, *et. al* highlights that the success of various cell-surface display systems cannot be predicted *a priori,* and surface display is best tested *in vivo* (Nicchi et al., 2021). Ice nucleation protein (INP) is a surface protein native to *Pseudomonas syringae* with N and C terminals separated by a varying number of spacer domains (Van Bloois et al., 2011). Previous studies have shown that the N domain alone can facilitate surface-display in *E. coli* with protein cargoes of varying sizes (Van Bloois et al., 2011). Therefore, we fused the N domain of INP with a truncated silicatein-α from *Tethya aurantia.* Recent work by Godigamuwa, *et al.* fuses silicatein-α with surface display protein *InaK* (often considered identical or analogous to INP), but rather than utilizing silicatein-α as a surface-displayed protein for *in vivo* activity, purifies the protein via established techniques (Godigamuwa et al., 2023). With the exception of the SDS-PAGE gel and Western blot to highlight protein expression, INP- silicatein-α was used *in vivo* for this work.

**FIGURE 2:**
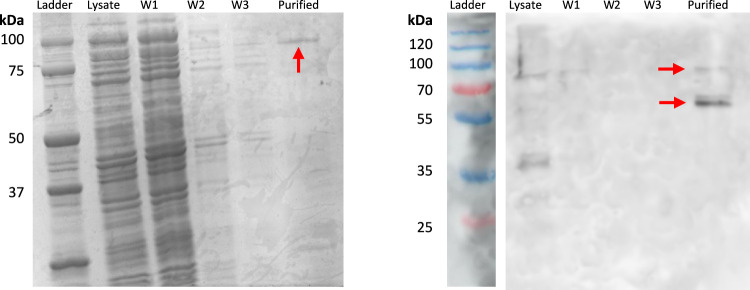
SDS-PAGE and Western blot highlighting INP-silicatein-α expression. SDS-PAGE for INP-silicatein-α purification with cell lysate, wash 1 (W1), wash 2 (W2), wash 3 (W3), and purified fractions. Western blot shows lysate, W1, W2, W3, and purified fractions. Red arrows indicate bands consistent with anticipated molecular weight of INP-silicatein-α (51.5 kDa) and INP-silicatein-α dimer (103 kDa).

INP-silicatein-α expression was confirmed with SDS-PAGE and Western blot (Figure 2), with bands at approximately 51.5 kDa and 103 kDa. The band at 103 kDa is consistent with protein dimerization, which is unsurprising given that the native purpose of INP (ice nucleation) relies on oligomerization of protein structure (Garnham et al., 2011; Hartmann et al., 2022). Importantly, protein purification will not be necessary for application of INP-silicatein-α in biocementation, as the surface-display enables access to the enzyme while also providing some measure of stability. Surface protein expression and accessibility without purification is advantageous by limiting post-processing steps required for application.

**FIGURE 3:**
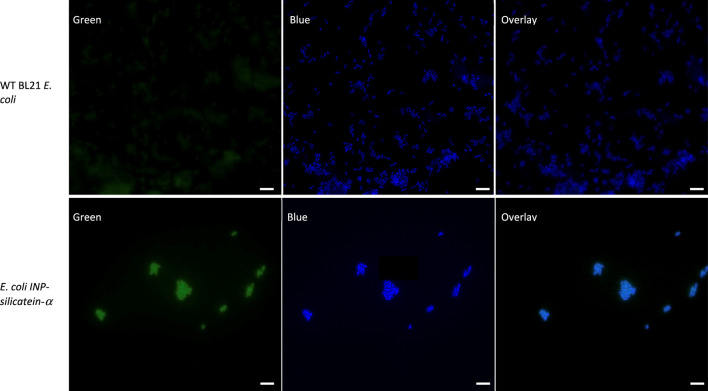
Immunocytochemistry of WT BL21 *E. coli* and *E. coli* INP-silicatein-α. Green fluorescence shows AlexaFluor 488 conjugated to anti-silicatein-α. Blue fluorescence shows DAPI staining of nucleic acids, highlighting the interior of bacterial cells. Overlay shows co-localization of INP-silicatein-α on the exterior of DAPI-stained cells for *E. coli* INP-silicatein-α, but not on WT BL21 *E. coli.*

To visualize INP-silicatein-α expression on the surface of the cells, we examined the cells with fluorescence microscopy following immunocytochemistry. An anti-silicatein-α antibody with AlexaFluor 488 was used to target silicatein-α expression on the surface of the cell. Membrane-permeable DAPI staining for nucleic acids was subsequently performed to highlight the interior of the cell. Figure 3 compares WT BL21 *E. coli* and *E. coli* INP-silicatein-α, illustrating DAPI staining in both samples, but green fluorescence only in cells expressing the INP-silicatein-α. These results verify that INP-silicatein-α is surface displayed and therefore accessible to substrates for biomineralization activity.

**FIGURE 4:**
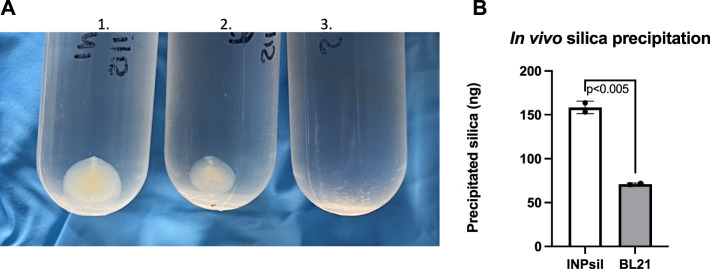
*In vivo* biomineralization with INP-silicatein-α. Silica mineralization can be visualized as precipitate after sample ultra-centrifugation. **(A)** 1) Precipitate from *E. coli* INP-silicatein-α and sodium orthosilicate after 24-h incubation, 2) precipitate from WT BL21 *E. coli* and sodium orthosilicate after 24-h incubation, 3) precipitate from sodium orthosilicate alone after 24-h incubation. **(B)** Comparison of precipitated silica (ng) as quantified via silicomolybdate assay. Two-tailed unpaired t-test (*p* < 0.005).

*In vivo* biomineralization was evaluated by introducing the common silica precursor sodium orthosilicate directly to *E. coli* INP-silicatein-α. Previous work shows direct biomineralization of inorganic silica precursors following incubation with silicatein-α over a period of 24 h at room temperature (Cha et al., 1999; Muller et al., 2008; Curran et al., 2017; Povarova et al., 2018). Following 24-h room-temperature incubation with silica precursor and subsequent ultra-centrifugation, mineralized silica was collected from *E. coli* INP-silicatein-α (Figure 4). Sodium orthosilicate precursor treated under the same conditions (without *E. coli* INP-silicatein-α) did not yield any precipitate. A quantitative measure of precipitate via silicomolybdate assay reveals that silica precipitation with *E. coli* INP-silicatein-α is significantly greater than with WT BL21 *E. coli* (unpaired t-test: t = 16.93, d.f. = 2, *p* = 0.0035), indicating that surface-displayed silicatein-α is biomineralization active. These results are promising for *in vivo* applications with surface-displayed silicatein-α, and effectively eliminates the need for protein purification, making biomineralization with silicatein-α less time and resource intensive.

Biocementation products such as bricks and columns are often used to measure strength and physical properties from the biocementation (Bachmeier et al., 2002; Zhao et al., 2014; Cardoso et al., 2020; Erdmann et al., 2022; Spencer et al., 2023). Compressive strength testing of cylindrical bricks made with *S. pasteurii* or *E. coli* INP-silicatein-α revealed encouraging results for the development of a silica-based biocementation pathway. As part of this work, WT BL21 *E. coli* treated bricks (without INP- silicatein-α expression) did not retain enough structure for unconfined compression testing, thus illustrating that *E. coli* alone does not have biocementation properties. (Non-cohesive soils like sand cannot be accurately measured in an unconfined compression test because they will immediately fall apart.) Similar results have been reported previously, highlighting the lack of urease activity and subsequent calcium carbonate precipitation associated with *E. coli* (Bachmeier et al., 2002; Liang et al., 2018; Heveran et al., 2019).

**FIGURE 5:**
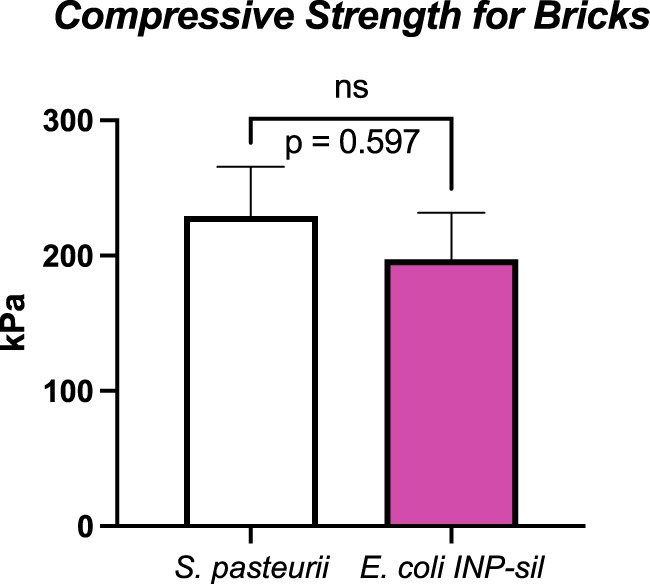
Unconfined compressive strengths for *S. pasteurii* and *E. coli* INP-silicatein-α bricks. *S. pasteurii* bricks show an average tolerance to 229 kPa (S.E.M. 36 kPa). *E. coli* INP-silicatein-α bricks show an average tolerance of 197 kPa (S.E.M. 34 kPa). A two-tailed unpaired t-test shows no significant difference between the two (*p* = 0.597).

*S. pasteurii* and *E. coli* INP-silicatein-α bricks withstood an average of 229 kPa (S.E.M. 36 kPa) and 197 kPa (S.E.M. 34 kPa), respectively (Figure 5). These compressive strengths are comparable to Type A soils, the most stable soil category as defined by OSHA (OSHA Technical Manual (OTM), 2014). Sand alone (*i.e.*, bricks prior to treatment with *S. pasteurii* or *E. coli* INP-silicatein-α), is considered a Type C soil, thus illustrating that the two different modes of bacterial biocementation explored here had significant impacts on soil cohesion. Furthermore, two-tailed unpaired t-test analysis, showed that the compressive strength of the two brick types did not differ significantly from each other (unpaired t-test: t = 0.5407, d.f. = 14, *p* = 0.59). The lack of a significant difference informs us that the two pathways yield comparable results and that a silica-centric biocementation pathway may be a viable option.

## Discussion

Notably, the cementation solution treatments containing urea and ammonium chloride may hinder *E. coli* viability and subsequent activity of INP-silicatein-α, thereby impeding silica formation. Higher concentrations of urea, such as in our cementation solution, have been shown to be toxic and inhibit growth of *E. coli* (Weinstein and McDonald, 1945; Schlegel et al., 1961; Taabodi et al., 2019). Alternatively, it is possible that the high levels of urea and ammonium chloride introduce porosity into the biomineralized silica structure (Kot et al., 2021), thereby weakening the biocementation product. In spite of this, cementation solution was applied to *E. coli* INP-silicatein-α as with *S. pasteurii,* to ensure that comparisons between the unconfined compressive strength could be credited to the different *E. coli* INP-silicatein-α and *S. pasteurii* microbes. Figure 5 shows that the unconfined compressive strength between bricks made with *S. pasteurii* and bricks made with *E. coli* INP-silicatein-α are not significantly different, highlighting the promising potential for INP-silicatein-α biosilicification in biocementation. We are currently testing to find the optimal conditions for biocementation with silica, including removing the urea and calcium cementation solutions in order to mitigate the production of harmful byproducts from traditional MICP.

The use of biocementation for cement production, improving soil strength, and dust mitigation is becoming increasingly possible with new and emerging research, with many studies applying biocementation techniques to stabilizing loose sediment via “grouting,” sealing undeveloped roads, encapsulating pollutants, preserving historical stone structures, and even CO_2_ fixation (Dhami et al., 2014; Anbu et al., 2016; Portugal et al., 2020). Biocementation may also be necessary in efforts to colonize other planets. One major roadblock for building structures on the moon or Mars is the cost of shipping tons of cement into space. *In situ* resource utilization strives to use the resources already present with minimal added materials. Implementing INP-silicatein-α expression in cyanobacteria, which research suggests can grow using only resources found on Mars and may thrive in space (Verseux et al., 2021; Mapstone et al., 2022; Ramalho et al., 2022), may be an avenue for biocementation, providing a necessary foundation for extraterrestrial construction at a relatively small cost. Lunar and martian regolith have calcium and silica content of approximately 14% and 49%, respectively (Exolith Lab, 2023), which suggests that biocementation via biosilicification may be a suitable target.

Additionally, the expression of a silica biocementation pathway in *E. coli* rather than *S. pasteurii* allows for future genetic engineering optimization and greater enhancement of the silica pathway. *E. coli* has proven to be an ideal platform host for development of industrially viable productions (Pontrelli et al., 2018). While *S. pasteurii* relies on a narrow pH range, more specific temperature, and precise urea concentrations for optimal biocementation, *E. coli* can be more easily genetically modified to adjust to different environmental conditions (Dong et al., 2023). Improvements and optimization of the silica biocementation pathway through *E. coli* can bring us one step closer to biomanufactured cement for space applications. Figure 5 shows that biosilicification with INP-silicatein-α is a promising alternative to biocementation with *S. pasteurii* as there is no significant difference between unconfined compression strength of bricks made with each. Upon optimization of INP-silicatein-α in *E. coli*, this system can be adapted for expression in cyanobacteria. Cyanobacteria may potentially provide numerous benefits in improvement of this system; they are photosynthetic, potentially providing an additional source of oxygen in extraterrestrial environment, and different strains have evolved to withstand more extreme conditions and stressors—high and low temperatures, desiccation, variable pH, and fluctuating salinity (Berla et al., 2013). The ability for cyanobacteria to survive and grow in hostile environments, combined with biosilicification activity, could make biocementation on distant planets a reality.

In the future, optimization experiments for biosilicification with *E. coli* INP-silicatein-α can assess silica mineralization with varying conditions and timescales. Further details such as any potential effects from local bacterial populations or potential effects from *E. coli* INP-silicatein-α survival in lab or field environments should be examined to further enhance the efficiency and long-term impacts of biocementation via biosilicification. These studies will lay the foundation for future biocementation applications and research.

## Data Availability

The original contributions presented in the study are included in the article/Supplementary material, further inquiries can be directed to the corresponding author.
